# Treatment of Experimental Cerebral Malaria by Slow Release of Artemisone From Injectable Pasty Formulation

**DOI:** 10.3389/fphar.2020.00846

**Published:** 2020-06-12

**Authors:** Jacob Golenser, Nadeen Salaymeh, Abd Alroof Higazi, Mohammed Alyan, Mahran Daif, Ron Dzikowski, Abraham J. Domb

**Affiliations:** ^1^Department of Microbiology and Molecular Genetics, The Kuvin Centre for the Study of Infectious and Tropical Diseases, Faculty of Medicine, the Hebrew University (HU), Jerusalem, Israel; ^2^Clinical Microbiology, the Hadassah Hospital, Jerusalem, Israel; ^3^Faculty of Medicine, School of Pharmacy, Institute of Drug Research, HU, Jerusalem, Israel

**Keywords:** cerebral malaria, artemisone, *Plasmodium*, slow release, pasty polymer formulation

## Abstract

Malaria caused by *Plasmodium falciparum* causes numerous cases of morbidity with about 400,000 deaths yearly owing, mainly, to inflammation leading to cerebral malaria (CM). CM conventionally is treated by repetitive administration of anti-plasmodial drugs and supportive non-specific drugs, for about a week. A mouse model of CM caused by *Plasmodium berghei* ANKA, in which brain and systemic clinical pathologies occur followed by sudden death within about a week, was used to study the effect of artemisone, a relatively new artemisinin, within an injectable pasty polymer formulated for its controlled release. The parasites were exposed to the drug over several days at a non-toxic concentrations for the mice but high enough to affect the parasites. Artemisone was also tested in cultures of bacteria, cancer cells and *P. falciparum* to evaluate the specificity and suitability of these cells for examining the release of artemisone from its carrier. Cultures of *P. falciparum* were the most suitable. Artemisone released from subcutaneous injected poly(sebacic acid–ricinoleic acid) (PSARA) pasty polymer, reduced parasitemias in infected mice, prolonged survival and prevented death in most of the infected mice. Successful prophylactic treatment before infection proved that there was a slow release of the drug for about a week, which contrasts with the three hour half-life that occurs after injection of just the drug. Treatment with artemisone within the polymer, even at a late stage of the disease, helped to prevent or, at least, delay accompanying severe symptoms. In some cases, treatment prevented death of CM and the mice died later of anemia. Postponing the severe clinical symptoms is also beneficial in cases of human malaria, giving more time for an appropriate diagnosis and treatment before severe symptoms appear. The method presented here may also be useful for combination therapy of anti-plasmodial and immunomodulatory drugs.

## Introduction

Malaria caused by *Plamodium falciparum* (Lee et al., 2019) is responsible for numerous cases of morbidity with about 400,000 deaths yearly (Weiss et al., 2019), mainly due to systemic inflammation (Coban et al., 2018) and damage to blood–brain barrier (Tunon-Ortiz and Lamb, 2019) leading to brain injury known as cerebral malaria (CM) (Ghazanfari et al., 2018). Conventional anti-plasmodial drugs and supportive non-specific drugs are used to treat human cases of CM (Golenser et al., 2006; Kyu and Fernández, 2009; Bruneel, 2019). Owing to the deleterious role of the immune system in the induction of CM and often cognitive limitations for life (Postels et al., 2019), it has been suggested that immunomodulation should be a part of the treatment of CM (Waknine-Grinberg et al., 2013; Dende et al., 2015). Therefore, optimal treatment for curing CM and limiting its risks should include both immunomodulatory and anti-plasmodial drugs. Owing to the systemic and potential chronicity of malaria (Ashley et al., 2018), treatments repeated over at least three days is recommended (Kyu and Fernández, 2009). Even so, disappearance of parasites from the blood stream does not necessarily indicate total cure as parasites may stay in the blood stream or hidden in the liver (Markus, 2017).

Blood dwelling parasites like plasmodia can be treated with artemisinins that act by radical production mediated by iron released from digested hemoglobin in the parasitized erythrocytes (Olliaro et al., 2001; Feng et al., 2019). However, this requires high doses of the drugs which are administered above the minimum inhibitory concentration affecting the parasites, and may cause systemic toxicity to patients (Schmuck et al., 2009; Devillers and Devillers, 2019). Moreover, the therapeutic effects of the drugs cannot be maintained for a long time because of pharmacokinetics and pharmacodynamics specifications (Alavijeh et al., 2005; Wright et al., 2011). Various controlled release drug systems, employing biodegradable carriers, have been suggested to prevent the risk of giving exaggerated doses. The advantages of using such carriers can be summarized as improvement in bioavailability, reduction of drug side effects (Beloqui et al., 2013) and increase in medication efficacy, (Kishimoto et al., 2016; Kumar et al., 2016; Charlie-Silva et al., 2018). Here, we used subcutaneous injections of a pasty polymer containing a known concentration of artemisone for treatment of experimental CM in a mouse model. A similar approach was applied successfully in treating mice infected with *Schistosoma mansoni* (Gold et al., 2017). The artemisone used in this study is a relatively new artemisinin derivative purported to be superior to the commercial artemisinins used currently (reviewed in Golenser et al., 2017). Like other artemisinins, artemisone demonstrates anti-inflammatory properties (Guiguemde et al., 2014; Lu et al., 2018). Resistance to artemisinins by plasmodia has been documented in many endemic areas (Tiwari and Chaudhary, 2020). There is no apparent reference to resistance to artemisone.

## Materials and Methods

### Artemisone in Gel

Artemisone was donated by CIPLA India. Artemisone in poly(sebacic acid–ricinoleic acid) (PSARA) gel was produced according to Haim-Zada et al. (2017) by combining ricinoleic acid (RA) with poly sebacic anhydride (PSA) and repolymerization of the mixture. PSA was prepared by reacting sebacic acid with acetic anhydride for 1 h under reflux, followed by solvent evaporation and polymerization under vacuum. Ricinoleic acid (70 g) was added to 30 g of the formed PSA and reacted at 160°C for 24 h under vacuum to yield RA-SA dicarboxylic acid oligoesters. These oligoesters were activated by acetic anhydride and polymerized for 4 h at 140°C under vacuum (10–15 mbar) to yield poly(RA-SA) 70:30 pasty injectable polymer. Different amounts of artemisone powder were mixed in the pasty polymer at 40°C and loaded in a syringe, ready for injection.

### *Plasmodium berghei* ANKA (PbA)

The strain of PbA (MRA-311, CDC, Atlanta, GA, USA) was maintained *in vivo* by serial transfer of parasitized erythrocytes from infected to naive mice. Plasmodial parasitemias in mice were estimated microscopically by determining the percent of parasitized erythrocytes in 5,000 counted erythrocytes seen in Giemsa stained thin smears of blood obtained from the tail veins of mice.

### Hematocrit Count

Blood was collected from the mouse tail vain in capillaries that were sealed and centrifuged (3,000 RPM in Hermle Z 231 M centrifuge). The erythrocyte percent was measured with an appropriate ruler.

### *P. falciparum* NF54 Culture

Parasites at a 5% hematocrit were cultured in RPMI 1640 medium supplemented with 0.5% Albumax II (Invitrogen, Carlsbad, California, United States), 0.25% sodium bicarbonate, 0.1 mg/mL gentamicin and 0.05 g/L hypoxanthine. Aliquots were divided to microplates (Nunc) at initial parasitemia of 0.5%. The parasites were incubated at 37°C in a gas mixture of 5% oxygen, 5% carbon dioxide and 90% nitrogen. Artemisone was added for 48 h, after which parasitemias were estimated using a microscope to examine thin blood smears of samples from individual wells, stained with Giemsa.

### Staphylococcus epidermidis (Gram^+^) and Escherichia coli 8957 (Gram^−^)

Bacteria were donated by the Department of Clinical Microbiology of the Hadassah Hospital, Jerusalem. Bacterial suspensions were prepared by dilution in LB growth media to an optical density (O.D) of 0.1 at 600 nm. The microplate was incubated at 37°C for 22 h, during which the O.D. was measured at 600 nm every 2 h using a spectrophotometer (UV–Vis detector, Tecan spark 10M instrument). Graphs of O.D. vs. time were used to determine the drug concentration that inhibits 50% of growth (ED50).

### HT-29 Human Colon Cells

HT-29 human colon cells were donated by Prof. Y. Barenholz from The Hebrew University. 20,000 cells were seeded in each well of a 96 well microplate in DMEM medium. Two days later the cells were incubated with artemisone for 48 h. Colorimetric XTT assay (Merck Guide; Beit Haemek, Biological Industries, Israel) was performed after an additional two days.

### THP1 Human Leukemia Monocytes

THP1 cells were grown in RPMI 1640 medium containing 10% fetal calf serum in flat bottom microplates kept at 37°C in an atmosphere of 5% CO_2_ in air. One day later Alamar Blue (Sigma, Israel) was added, and 24 h later the resulting fluorescence was measured using a microplate reader (Fluoroskan Ascent F1, Finland; Keurulainen et al., 2015).

### Bioassay for *In Vitro* Release of Artemisone From the Polymer

Released artemisone was quantified in a bioassay using two-day cultures of artemisone- sensitive *P. falciparum* (Gold et al., 2017). Briefly, polymer samples containing 2 mg of artemisone were sterilized by UV exposure and then each sample was transferred to 1 ml RPMI 1640 medium in each well of 24-well flat bottom disposable sterile plates (Nunc). The plates were incubated at 37°C in an atmosphere of 5% CO2 in air. Once each day the medium was collected and frozen until use. The polymers were washed twice in 2 ml of fresh medium and 1 ml medium was added, and the plates were returned to the incubator. Different dilutions of the collected supernatants were examined for growth inhibition of *P. falciparum*. This was done in flat bottom 96-well plates (Nunc A/S, Roskilde, Denmark). Parasitemias were estimated as described above.

### Mice

Male C57BL/6 mice were purchased from Harlan Laboratories (Jerusalem, Israel) and used to generate and maintain PbA infections for experimentations.

### Treatment of Mice

Artemisone in gel at 0.1 or 0.2 ml per dose was injected SC into the abdomens of infected mice at different days before or after infection. The animal study protocol was approved by The Hebrew University Institutional Animal Care and Use Committee (Protocol No. MD-12-1351; Golenser’s accreditation No. 12180). All procedures followed the institutional guidelines.

### Induction of Experimental CM (ECM)

C57BL/6 male mice (7-8 week old) were infected by intraperitoneally injecting 5 × 10^4^ parasitized erythrocytes from peripheral blood of PbA infected donor mice. This inoculum caused fatal experimental CM (ECM) in at least 80% of the infected mice. The link between early death and ECM in mouse models has been discussed previously (Waknine-Grinberg et al., 2010); mice that died at a parasitemia of 20% or below with accompanying neurological symptoms and drastic reductions in body weight and temperature died of ECM. Untreated mice that were resistant to ECM succumbed later to severe anemia and hyperparasitemia (Engwerda et al., 2005).

### Blood–Brain Barrier Damage

Mice were infected by PbA and treated 3 days post-infection by subcutaneous artemisone in gel (1 mg ART/0.1 ml gel). Evans Blue dye was injected to the tail vein (0.2 ml of the dye, 2% in PBS) in day 7 post-infection to assess damage to the blood–brain barrier. After 1 h the mice were anesthetized, perfused with PBS and the brains were removed and photographed.

### Liver Functions

Mouse blood was separated and centrifuged in Minicollect 1 ml tubes. Serum was separated after centrifugation at 3,000*g* for a period of 10 min in 15 °C and diluted 1/3 to reduce hemoglobin interference. Alanine transaminase (ALT), Aspartate transaminase (AST) and Alkaline phosphatase (ALK) were analyzed on the day of sampling using Cobas 6000 analyzer c501 module. About 200 µl of each sample were inserted into the analyzer for automatic chemical colorimetric analysis of all enzymes.

### Cytokine Assay

Enzyme-linked immunosorbent assay (ELISA) kits for murine plasma cytokine analysis were purchased from BioLegend, Israel. C57BL/6 mice were injected with PbA and treated with artemisone in polymer at different days post-infection. Later, mice were sacrificed, blood samples were collected in MiniCollect tubes (Greiner Bio-One, Germany) and serum was separated after centrifugation. Serum cytokine analysis of IL-10, IFN-γ, IL-4, and TNF by ELISA, was conducted according to the manufacturer’s instructions.

### Statistics

Experiments were repeated at least once. Experimental groups were compared to control groups using Student’s t-test.

## Results

### Specificity Tests

*P. falciparum*, *S. epidermidis*, *E. coli 8957*, *THP-1* human monocytes and *HT-29* human colon cells were examined for their sensitivity to artemisone and artesunate and possible suitability in a bioassay for artemisinins (**Table 1**). Despite the ease of culturing the bacteria and the human cells compared to plasmodia, they cannot be used for a bioassay because of their very low sensitivity to the drugs. Consequently, the following bioassay for artemisone release was performed by using *P. falciparum* cultures. Apparently, the human cells were more sensitive to artesunate than to artemisone, and the opposite was recorded for *P. falciparum*.

**Table 1:** Specific and non-specific *in vitro* effects of artemisone.

Cells	Artemisone ED50	Artesunate ED50
*P. falciparum*	1–3 ng/ml	8–12 ng/ml
*S. epidermidis*	Above 5 µg/ml	Above 5 µg/ml
*E. coli 8957*	Above 5 µg/ml	Above 5 µg/ml
*THP-1* human monocytes	174 µg/ml	67 µg/ml
*HT-29* human colon cells	Above 100 µg/ml	70 µg/ml

### *In Vitro* Release of Artemisone

A mix of 2 mg artemisone in 0.1 ml pasty polymer was attached to the bottom of each well of 24 well plates (Nunc) and 1 ml of medium was added to each well. Dilutions of supernatants collected daily from the wells were examined *in vitro* for activity against *P. falciparum*. Supernatants from incubated gels collected after 3 and 7 days at a dilution of 1/62,500 reduced parasitemias by 98 and 75%, respectively. There was no significant activity in supernatants collected from wells that contained gels without the drug. Free artemisone had an ED_50_ at a concentration of, approximately, 2 ng/ml. The results indicate marked *in vitro* release of artemisone at least within a week.

### Treatment of Infected Mice

In preliminary experiments, injection of just polymeric gel or just its individual components at various times either before or after infection with PbA had no effect on the course of the infection and all treated and untreated mice died of CM within 9 days post-infection.

#### Prophylactic Treatment

**Figure 1:**
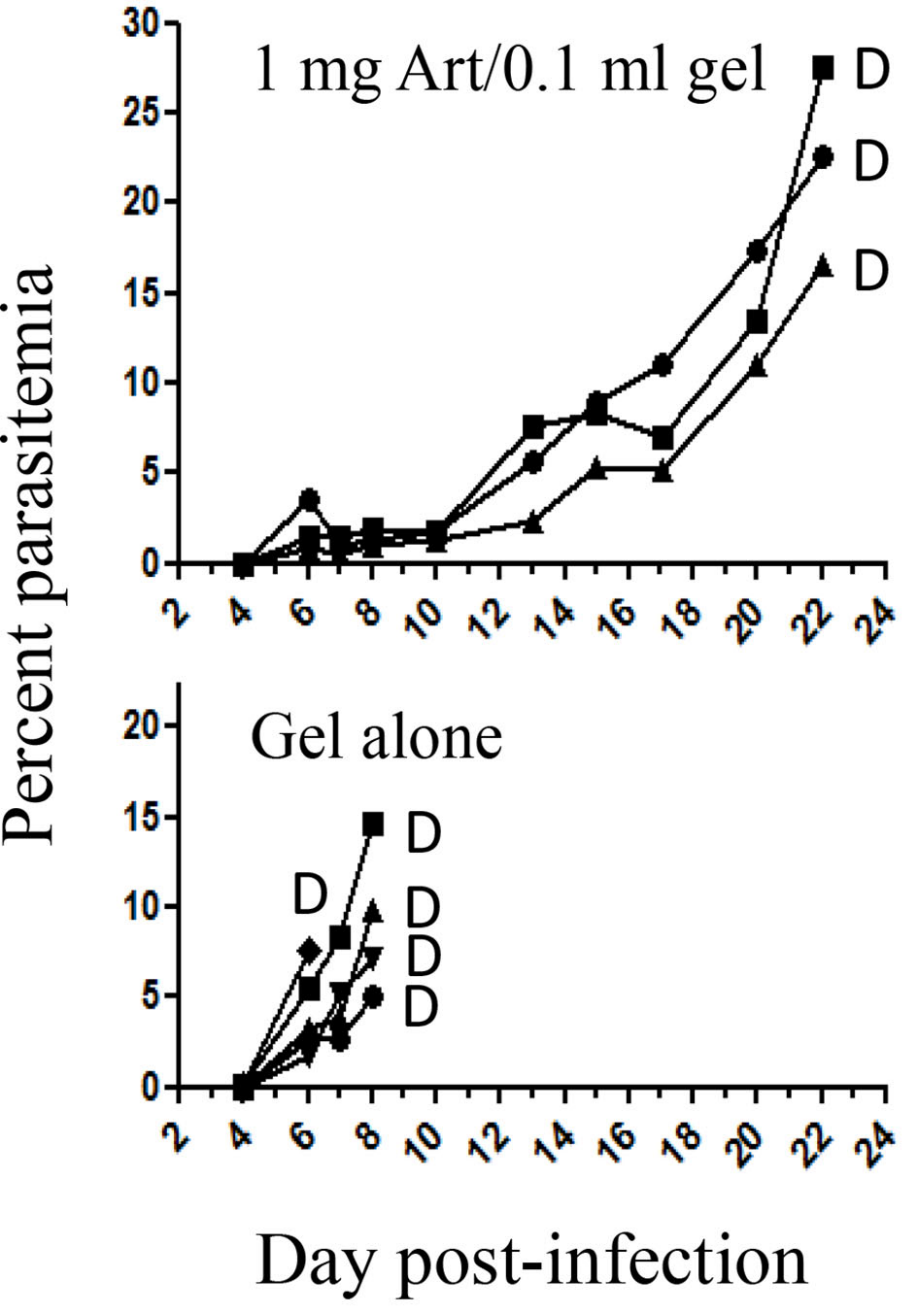
The effect of prophylactic treatment at four days before infection with PbA. Each curve represents one mouse; Art, artemisone; D, death; Gel alone n = 5; Treated n = 3.

*In vivo* prophylactic treatment was by injecting a single SC dose of artemisone 1 mg/0.1 ml gel, 4 days before infection with PbA. All control mice died within eight days post-infection when parasitemias were relatively low. This result indicates death of CM in this model of murine malaria. In contrast, the mice in the treated group died 2 weeks later of anemic malaria (**Figure 1**). This proves that 4 days after drug delivery there was enough circulating artemisone to modify the course of the disease.

#### Hematocrit Values of Treated Mice

Hematocrits of infected mice treated with gel alone, dying of CM when parasitemia was relatively low, were normal (**Table 2**). The treated mice died of anemia about two weeks later (in correlation with the results that are presented in **Figure 1**).

**Table 2:** Hematocrit values of treated mice measured 2 days before their death.

Group (n = 5 mice/group)	Day post-infection	Average hematocrit ± standard error
Uninfected	–	48 ± 1
Untreated (gel alone) infected	6	52 ± 2
Treated infected*	20	19 ± 2

P = < 0.01 for treated vs untreated infected and uninfected mice; there was no difference between the untreated infected and the uninfected ones.

#### Treatment Post-Infection

**Figure 2:**
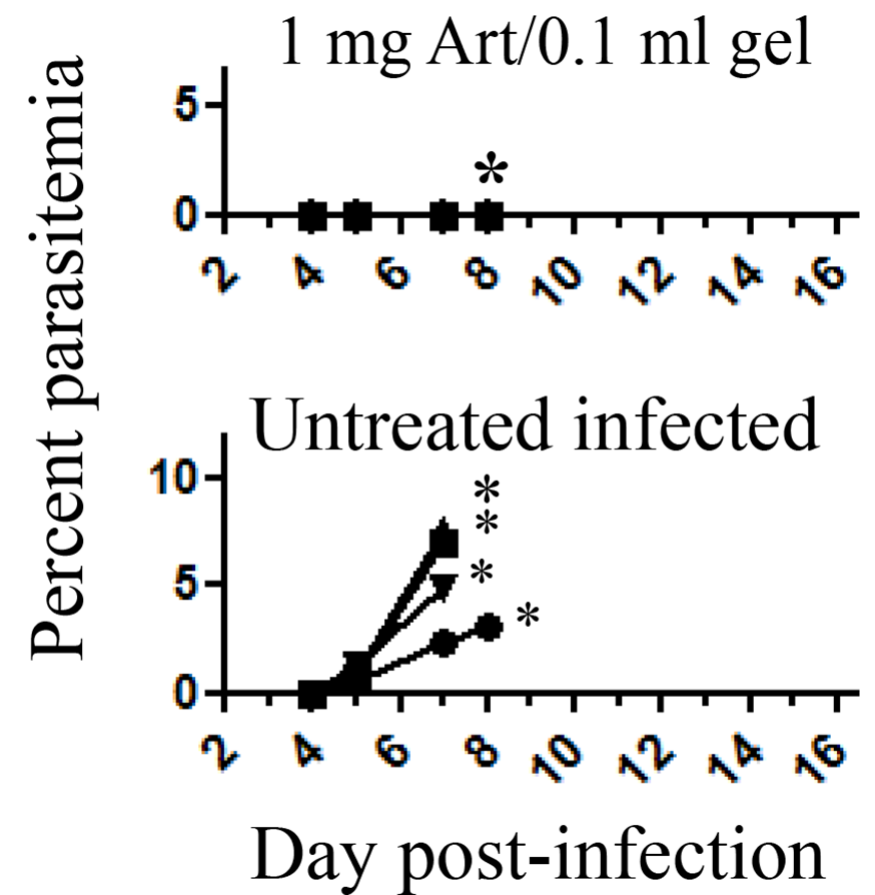
The effect of treatment at 2 days post-infection with PbA. Each curve represents one mouse; Eight days post-infection treated* and untreated* mice that were about to die of CM P = <0.001; were sacrificed to estimate liver function; Untreated infected n = 4; Treated n = 6, all treated mice that had not been sacrificed survived; Art, artemisone.

Mice were injected with a single SC dose of artemisone 1 mg/0.1 ml gel at 2 days post-infection. Untreated mice were dying of CM and sacrificed for estimating their liver functions. Treated mice cleared the parasites but some were sacrificed for the same purpose (**Figure 2**).

Early treatment completely suppressed parasitemia. In similar experiments the control mice died within nine days post-infection, and treated mice did not demonstrate parasitemias and any disease symptoms throughout one month follow up.

**Figure 3:**
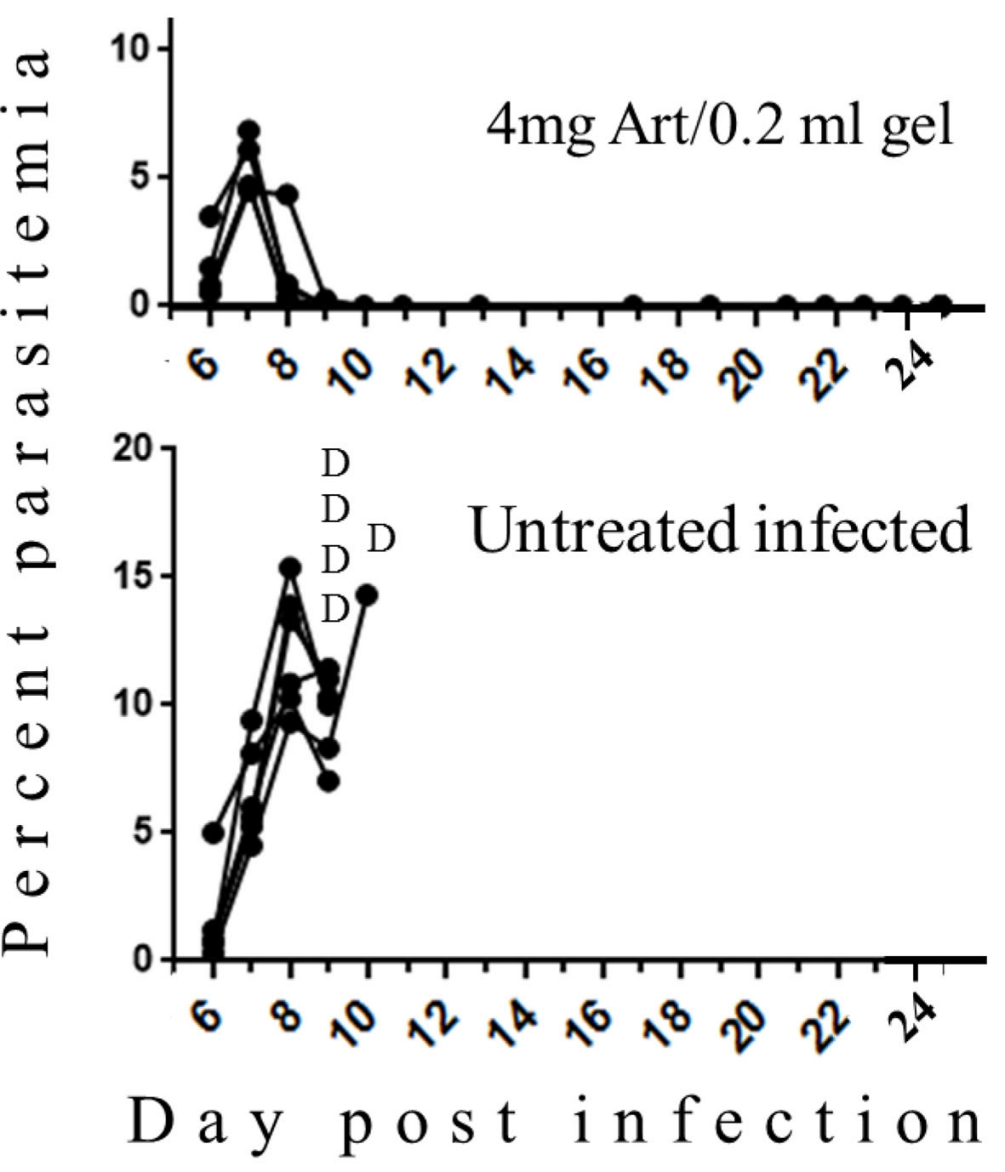
The effect of late treatment at five days post-infection with PbA. Each curve represents one mouse; Art, artemisone; D, death; Untreated n = 5; Treated n = 4, all survived.

Late treatment that was performed at 5 days post-infection when symptoms of CM were obvious totally reduced parasitemias and saved all treated mice. All control mice died of CM (**Figure 3**). Despite using increased amounts of artemisone, there were no signs of side effects.

### Blood–Brain Barrier After Treatment

Infected mice were treated at day 3 post infection. Four days later Evans Blue was injected and brains were examined.

**Figure 4:**
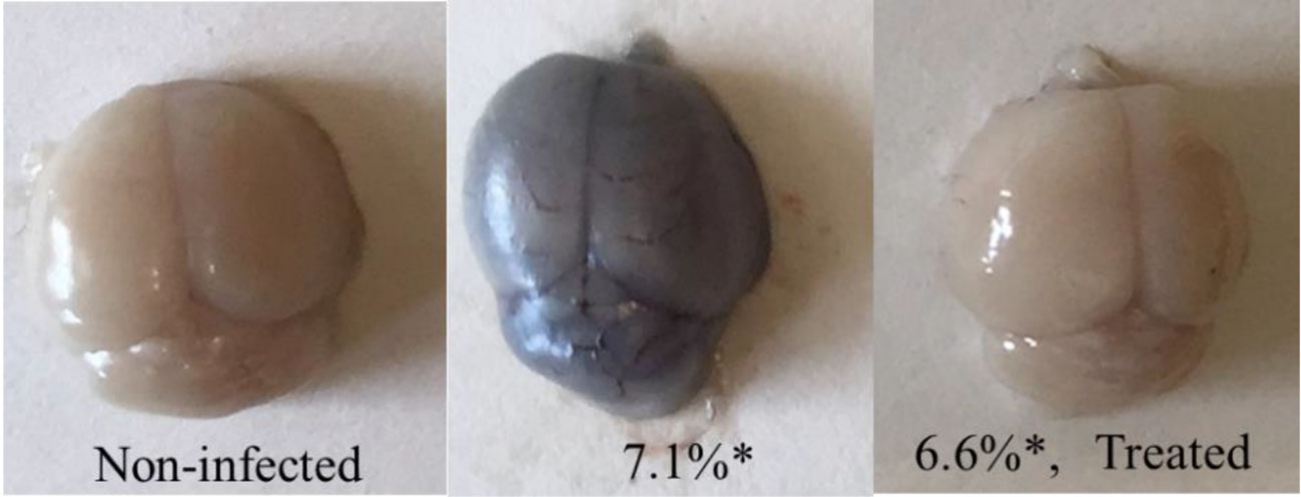
The effect of treatment at three days post-infection on brain infiltration of Evans Blue. Brain examinations were conducted seven days post infection. *Parasitemias at the day of brain examinations. n = 3/group.

Representative photographs (**Figure 4**) demonstrate the positive effect of the treatment in counteracting the destruction of the BBB as reflected by lack of Evans Blue infiltration into the brains of treated mice.

### Liver Function After Treatment

Mice were treated 4 days post-infection and three days later were examined for liver function. The infection decreased ALK and elevated ALT and AST levels in comparison with uninfected mice (p <0.01). Three days after the treatment ALT and AST levels returned to normal levels (no significant difference) but ALK level of the treated mice was still as low as that of the untreated mice (**Table 3**). Later, ALK levels also returned to normal (**Table 4**).

**Table 3:** The effect of treatment on liver functions of treated, and untreated infected mice.

Group	Mean ± standard error [u/L]		
	ALK	ALT	AST
Treated infected	50 ± 3.5	43 ± 11.8	122 ± 4.5
Untreated infected	63 ± 1.5	381 ± 76	626 ± 67
Uninfected	158 ± 10	57 ± 12	151 ± 25

Artemisone in gel treatment

ALK, Alkaline phosphatase; ALT, Alanine transaminase; AST, Aspartate transaminase. n = 3/group.

**Table 4:** The effect of treatment on liver function of convalescent mice.

Group	No. mice	Mean ± standard error [u/L]		
		ALK	ALT	AST
Treated infected	4	136 ± 11	37 ± 2	115 ± 8
Uninfected	3	155. ± 12	39 ± 1	116 ± 9

ALK, Alkaline phosphatase; ALT, Alanine transaminase; AST, Aspartate transaminase.

Mice were treated with artemisone in gel at day 4 post-infection and liver function assessment was done 14 days later. The treated mice had normal levels of liver functions; there were no significant differences in all liver functions between the treated mice and the uninfected mice. All infected untreated mice died within eight days post-infection (see **Figure 2**).

### Cytokines After Treatment

Serum was separated as described in *Materials and Methods*. Treatment was performed at day 4 post-infection and serum samples were collected for cytokine assessment 3 days later (**Table 5**). In the untreated infected mice there was an increase in inflammatory cytokines interferon, and TNF relatively to the uninfected ones (p ≤0.01). There was a decrease (p < 0.01) or insignificant elevation in the anti-inflammatory ones IL-4 and IL-10, respectively. The treatment did not prevent the increase of interferon levels but there was a significant reduction in the TNF levels and an elevation in the anti-inflammatory cytokines, IL-4 and IL-10 compared to the levels of the untreated infected mice.

**Table 5:** Cytokine levels in mice treated with artemisone.

Group	No. mice	Percent parasitemia	IL-4 [pg/ml]	IL-10 [pg/ml]	TNF [pg/ml]	IFN [pg/ml]
Untreated infected	6	3.6 ± 1.2	BDL*	67 ± 11	727 ± 65	794 ± 84
Treated infected	6	0	27 ± 3	404 ± 35	232 ± 18	1026 ± 83
Uninfected untreated	4	–	51 ± 6	23 ± 3	60 ± 11	112 ± 9

*BDL, below detection level.

## Discussion

Human malaria is a systemic inflammatory disease causing physiologic, metabolic and immune abrogation, leading, for example, to cerebral symptoms, respiratory stress and sepsis, depending on the species of *Plasmodium* and genetics and immune status of the patients (Wassmer and Grau, 2017; Nishanth and Schlüter, 2019). Currently, malaria is treated by a limited number of drugs. The first line of treatment is artmisinin combination therapy (ACT) (Ashley et al., 2018). Treatment of CM, the most dangerous symptom, is liquid quinine (Nishanth and Schlüter, 2019) and supportive measures (Idro et al., 2010). Recently, artesunate has also been used for treating CM (Shah et al., 2020). Unfortunately, *P. falciparum*, the most dangerous species, of *Plasmodium* has become resistant to all anti-plasmodial drugs including ACTs (Tiwari and Chaudhary, 2020). Even so, current recommended treatment is still based on ACTs, except for infection during the first trimester of pregnancy, mainly because of potential neurotoxicity (Nontprasert et al., 2000; Schmuck et al., 2009).

Here, a mouse model with an infection caused by *Plasmodium berghei* ANKA was used to represent severe malaria. The mice die within about a week of what is considered to be CM and can be used as a valid model imitating human CM (Hunt et al., 2010; Varga et al., 2010). The aim was to achieve slow release of artemisone from a pasty gel to prevent CM and alleviates other symptoms of the infection. Artemisone is considered to be less toxic than other artemisinins and has longer *in vivo* half time probably because of reduced first pass metabolism (Haynes et al., 2006). The common dogma is that artemisinins affect plasmodia by reacting with iron (available in the parasitized erythrocyte) to produce lethal artemisinin radical. This is challenged by Wong et al. (2020) who found a strong effect of artemisone on other stages of the parasite that have no heme metabolism. Haynes et al. (2003) proposed that “iron acts as a Lewis acid to facilitate ionic, rather than radical, bio-activation of the artemisinin” (reviewed by Kumari et al., 2019). Haynes and co-workers also attribute the effect to redox imbalance (Wong et al., 2020). In both *in vitro* and *in vivo* experiments artemisone was found to be more effective than artesunate which is the most commonly used artemisinin (Ramharter et al., 2006; Waknine-Grinberg et al., 2010). Here, the effect of both agents on various cells was examined and, accordingly, the more effective artemisone was inserted into injectable pasty polymer, enabling a controlled release without burst (Pawar et al., 2010; Gold et al., 2017).

Artemisone affected the *P. falciparum* with ED50 of a few ng/ml, an amount much lower than that was needed to affect bacteria and animal cells. This correlates with the results of Wong et al. (2020) who used CHO cells and Oiknine-Djian et al. (2018) who used CMV. Artemisone was more active than artesunate, confirming earlier findings. The results should be considered in the context of artemisone’s mode of action where parasites are blood dwelling and exposed to increased heme initiated formations of free radicals (Saeed et al., 2016; Gold et al., 2017). Hence, there is a difference between higher sensitivity of plasmodia and schistosomes to artemisone compared to the animal cells, bacteria, and virus.

Slow release of artemisone from the pasty polymer was confirmed *in vitro* using a bioassay based on sensitivity of *P. falciparum*, and *in vivo* in mice infected with PbA. *In vitro*, there was a significant release during at least a week. Taking into account that the T1/2 of artemisone *in vivo* is about 3 h, the estimated amount of free artemisone theoretically, four days later would be negligible. However, this amount of artemisone (up to 3 mg) if injected directly into mice, would immediately kill the mice. In contrast, the *in vivo* prophylactic injection of the polymer 4 days before infection prevented CM. Early and late treatments 2 and 5 days post-infection, respectively, abolished the parasitemia, and prevented the lethal CM that occurred in the untreated control mice. There were no gross signs of toxicity when the drug was administered within the polymer. Neither the polymer nor its individual components had any effect on the course of the disease.

In human cases initiation of CM is attributed to a breakdown in the blood–brain barrier (BBB) (Dende et al., 2017). Here, symptoms of CM and eventual death were also correlated with destruction of BBB and successful treatment was expressed in both prevention of BBB damage and recovery of infected mice. No parasites were found in the brains of treated mice and few parasites were found in the brains of the untreated mice (data not shown).

Cytokine examinations reflected the inflammatory nature of the disease. Following infection, inflammatory cytokines were elevated while anti-inflammatory cytokines were reduced. The treatment with artemisone reversed these phenomena, possibly owing to the reduction in the number of parasites, but also because of the action of the anti-inflammatory mechanism attributed to artemisinins (Ho et al., 2014; Guiguemde et al., 2014).

The systemic nature of malaria in humans is known and various pathologies may accompany CM (Krishnan and Karnad, 2003). In parallel, malaria affects different organs in experimentally infected mice (Craig et al., 2012). In severe infections of malaria caused by *P. falciparum* among humans, involvement of the liver is common and significant cause of morbidity and mortality (Viriyavejakul et al., 2014). Likewise, in malaria research, rodent models have been pivotal for studying liver syndromes that follow malaria infection (De Niz and Heussler, 2018; Edagha et al., 2019). Notably is the notion that the liver stage of plasmodium is a critical checkpoint for development of CM (Sato et al., 2019). In accordance with these studies we found in the infected mice, clinical effects that were expressed as changes in hepatic enzymes, i.e. elevated ALT and AST and decreased ALK. Treatment of the mice with artemisone resulted in a return to normal levels of these hepatic enzymes in parallel with the general recovery of the animals.

Overall, an anti-malarial curative effect was achieved by the slow release of artemisone in an injectable pasty polymer that prevented CM. This could serve in producing a cure against the most severe form of malaria and taken as a response to the “Desperately seeking therapies for cerebral malaria” of Riggle et al. (2020). The method researched here might also be used for other drugs and diseases. It might also increase compliance among sick people needing long term and repetitive treatments.

## Data Availability Statement

The raw data supporting the conclusions of this article will be made available by the authors, without undue reservation, to any qualified researcher.

## Ethics Statement

The animal study was reviewed and approved by The Hebrew University Institutional Animal Care and Use Committee (protocol No. MD-12-1351; Golenser’s accreditation No. 12180).

## Author Contributions

JG and AD planned the experiments and participated in the experimental work. MA and MD produced the gels. NS examined malaria development in mice and drug effects on bacteria. AH measured liver functions. RD detected drug effects in *P. falciparum* cultures. AD, RD, and JG wrote the paper.

## Funding

The authors are thankful to the Deutsche Forschungsgemeinschaft (DFG) that sponsored the trilateral German-Israel-Palestine (GIP) project (GR 972/47-2).

## Conflict of Interest

The authors declare that the research was conducted in the absence of any commercial or financial relationships that could be construed as a potential conflict of interest.
